# Hearing loss in HIV-exposed but uninfected children: Advocating for the role of audiologists in South Africa

**DOI:** 10.4102/sajcd.v72i1.1084

**Published:** 2025-03-12

**Authors:** Katijah Khoza-Shangase

**Affiliations:** 1 Department of Audiology, Faculty of Human and Community Development, University of the Witwatersrand, Johannesburg, South Africa

**Keywords:** HIV, hearing loss, audiology, early hearing detection and intervention, antiretroviral therapy, HIV-exposed uninfected children, ototoxicity, South Africa

## Abstract

Hearing loss in children born to human immunodeficiency virus (HIV)-positive mothers is an emerging public health concern in South Africa. These children, particularly those exposed to antiretroviral therapy (ART) in utero, face heightened risks of auditory impairment predominantly linked to ototoxic ART regimens, prematurity, maternal health comorbidities such as diabetes, and socioeconomic vulnerabilities. While ART has been effective in reducing mother-to-child HIV transmission, evidence suggests that it can also contribute to auditory impairments, impacting early childhood development, language acquisition, and cognitive skills. Audiologists play a crucial role in mitigating these risks through early hearing detection and intervention (EHDI). However, audiologists remain underutilised by both the public and the National Health Department in public healthcare settings with capacity versus demand challenges, and routine audiological screenings for high-risk children, such as HIV-exposed but uninfected infants, are limited.

**Contribution:**

This article advocates for the integration of routine audiological assessments within maternal and paediatric HIV healthcare services, highlighting the need for structural changes in policy to support preventive audiological care. By establishing comprehensive, multidisciplinary EHDI programmes, South Africa can reduce the long-term developmental and socioeconomic impacts of hearing loss in this vulnerable population, enhancing both individual and public health outcomes.

## Introduction

Hearing loss among children born to HIV-positive mothers presents a significant, under-recognised public health concern in South Africa. With a high prevalence of HIV in South Africa (Human Science Research Council [HSRC], 2023), children born to HIV-positive mothers, especially those exposed to antiretroviral therapy (ART) in utero, face an increased risk of hearing impairments (Khoza-Shangase, 2021; Khoza-Shangase & Nesbitt, 2023; Padilha et al., 2018; Torre et al., 2012). Hearing loss in early childhood is associated with lasting effects on language, cognitive and psychosocial development, highlighting the critical role of audiologists in mitigating these impacts (Mushtaq et al., 2024). Audiologists are often excluded from multidisciplinary teams in maternal and paediatric healthcare, despite their critical role in early hearing detection and intervention (EHDI). In South Africa, this underutilisation is compounded by the well-documented manpower capacity/demand challenge where there are limited audiologists for the size of the population (Pillay et al., 2020). This oversight leads to missed opportunities for early diagnosis and intervention in high-risk populations. Sufficient evidence emphasises the importance of EHDI and the unique vulnerabilities of HIV-exposed but uninfected (HEU) infants (Khoza-Shangase, 2021). HIV-exposed but uninfected children often exhibit higher rates of auditory deficits because of the combined effects of in utero ART exposure, socioeconomic disparities and limited access to comprehensive maternal healthcare. This intersection of factors is particularly pronounced in South Africa, where healthcare inequities compound the challenges faced by HIV-positive mothers and their children. Despite this, there remains a concerning gap in routine audiological assessments for these children, particularly in South African public healthcare. The urgent need for audiologists’ involvement in the routine care of children born to HIV-positive mothers is essential, given the multifaceted risks these children face.

## Understanding the risks: HIV, antiretrovirals and hearing loss

The intersection of HIV exposure and the use of ART in mothers significantly increases the likelihood of hearing issues in their offspring (Padilha et al., 2018; Torre et al., 2012, 2016). While ART has been instrumental in reducing mother-to-child transmission (MTCT) of HIV to below 5% when administered effectively (Artz et al., 2020; Avert, 2018), some antiretroviral drugs have ototoxic (ear-damaging) side effects that can affect the developing auditory system in utero (DeBacker et al., 2022; Fasunla et al., 2018). Nevirapine (NVP), a key component of ART regimens for preventing MTCT, has been associated with potential ototoxic effects, particularly with prolonged exposure. While its efficacy in reducing HIV transmission is well-documented, studies by Fasunla et al. (2018) and Torre et al. (2012) highlight the need to monitor its long-term impact on the auditory systems of HEU children. Nevirapine-related toxicity may exacerbate sensorineural hearing loss by affecting cochlear structures, underscoring the importance of integrating ototoxicity monitoring into ART protocols. Research led by Khoza-Shangase and Nesbitt (2023) has shown a higher incidence of auditory deficits in HEU neonates than in HIV-unexposed peers. For instance, HEU neonates tested in a Johannesburg hospital presented with refer findings on both Distortion Product Otoacoustic Emissions (DPOAE) and Automated Auditory Brainstem Response (AABR) tests at rates significantly higher than those of HIV-unexposed neonates. Similarly, Torre et al. (2012) highlighted that perinatally HEU children are more likely to present with hearing loss because of in utero exposure to ART. These findings align with Padilha et al. (2018), who documented a higher prevalence of sensorineural hearing loss in HEU infants compared to their HIV-negative counterparts. Studies conducted in South African settings (Khoza-Shangase & Nesbitt, 2023) also highlight the disproportionately high rate of refer results in neonatal hearing screenings among HEU infants, further emphasising their classification as a high-risk group for auditory impairments.

In a recently completed retrospective review study on hearing function in children born to HIV-positive mothers in Johannesburg (submitted, 2024), overall findings revealed a hearing loss prevalence of 16.5% among children born to HIV-positive mothers, with bilateral impairment being the most frequently observed. The degree of hearing loss varied from mild to profound, with audiological evaluations largely indicating sensorineural impairment. Analysis of maternal health risk factors showed that ART regimens – especially those including protease inhibitors – and maternal diabetes were linked to higher likelihoods of hearing loss in these children. The findings demonstrate significant associations between specific ART regimens, maternal health conditions and hearing outcomes, highlighting key risk factors that may influence auditory development in this group. These findings indicate a pressing need for targeted EHDI programmes that include regular hearing assessments for HEU infants, particularly those exposed to ototoxic ART.

Furthermore, HIV-positive mothers often face complex socioeconomic barriers that exacerbate health risks for their children (HSRC, 2023; Rafaqat et al., 2024). Issues such as poor access to prenatal care, maternal malnutrition and limited awareness about the importance of hearing assessments compound the risk factors for hearing loss among HEU children. As children in these circumstances are more susceptible to developmental delays if hearing loss goes undetected, proactive audiological care becomes a critical aspect of preventive healthcare.

## Audiologists’ role in addressing a public health crisis

Given the high rates of HIV in South Africa and the specific vulnerabilities of HEU children, routine audiological monitoring should be a standard practice. Audiologists are uniquely trained to detect early signs of hearing impairment and can provide crucial interventions that mitigate the developmental consequences of hearing loss. However, audiologists are often underrepresented in neonatal and paediatric healthcare teams, particularly within South Africa’s public health system, where resources are scarce, and priorities are driven by the immediate, visible symptoms of illness rather than preventive care (Khoza-Shangase, 2022). To contribute meaningfully to their teams, audiologists should also engage in collaborative research with infectious disease specialists, pharmacologists and public health professionals. This would not only allow them to stay abreast of the latest developments in HIV treatment but also enable them to advocate for evidence-based audiological practices tailored to the needs of HEU children. Regularly updating clinical guidelines and leveraging digital tools, such as online databases tracking ART advancements and their side effects, can further enhance their role in addressing hearing loss within this dynamic healthcare landscape.

## The case for routine early hearing detection and intervention programmes in high-risk populations

Despite the availability of ART, the complex interaction between HIV exposure and ART ototoxicity necessitates a stronger, more standardised EHDI protocol. Routine screening is critical for children at risk, as early hearing loss – if left untreated – can severely impact speech and language development, academic performance and long-term quality of life (Kanji, 2022). Diagnostic data from international and local studies strongly advocate for prioritising HEU children in EHDI programmes, where early identification of auditory deficits in this population suggests an urgent need for intervention during the critical early developmental period. McDaid et al. (2021) emphasise the cost-effectiveness of targeted hearing screenings in resource-constrained environments, particularly for high-risk groups such as HEU children, where early diagnosis can prevent cascading developmental challenges. Data from studies such as those by Casoojee (2024), Casoojee et al. (2024) and Maluleke et al. (2021) highlight the effectiveness of early detection and intervention in preventing such outcomes, advocating for consistent screening to identify hearing loss before it disrupts developmental milestones. Given the unique, multi-layered risks faced by HEU children, South Africa’s healthcare policies must recognise hearing health as an essential component of postnatal care for these high-risk populations. Lessons can be drawn from established audiological monitoring programmes for children receiving ototoxic chemotherapy for cancer. These programmes, commonly found in high-income settings, emphasise proactive surveillance through baseline and serial audiological assessments, ensuring early detection of auditory damage. Unlike HIV-related EHDI programmes, which are often limited by resource constraints and inconsistent follow-ups, cancer-related programmes employ standardised protocols, multidisciplinary collaboration, and robust data tracking to guide treatment decisions and interventions (L’Hotta et al., 2023). By integrating similar principles, such as regular monitoring and early intervention, EHDI programmes for HEU children could achieve greater consistency and effectiveness in preventing long-term auditory deficits.

Integrating audiological screenings as a standard component of postnatal care would benefit not only HIV-positive mothers and their children but also public health outcomes. Early identification and timely interventions are cost-effective, reducing the long-term financial and social burdens associated with hearing loss. The benefits of these early interventions are especially relevant in resource-constrained settings, where untreated hearing loss can have long-term consequences for social inclusion, education and employment (Maluleke, 2022; McDaid et al., 2021).

## Advocating for policy and structural changes to support audiologists

To implement effective EHDI programmes, structural changes must be made at policy and healthcare systems levels. Five key changes are advanced in this article. Firstly, *integrating audiological services in paediatric HIV care* should be prioritised. Hospitals and clinics serving HIV-positive mothers and HEU children should have access to audiologists and appropriate equipment for regular hearing screenings, and where task-shifting can be done, appropriate resources and support be provided. Audiological assessments should be prioritised within maternal and paediatric health services, particularly at district-level facilities. This can be achieved by establishing district-level audiology programmes linked to paediatric HIV clinics and leveraging mobile health units for hearing screenings in underserved areas. Secondly, *establishing training programmes for multidisciplinary teams* should form part of national department of health strategic planning. Training for healthcare providers, including paediatricians, nurses, and HIV counsellors, is essential to build awareness about the risks of hearing loss in HEU infants and the importance of early detection. Given the dynamic nature of HIV treatment regimens, audiologists must engage in continuous professional development to stay informed about emerging ART protocols and their potential ototoxic effects. This could include attending interdisciplinary workshops, participating in HIV-focused medical conferences and maintaining active memberships in professional organisations that address ototoxicity in ART. Furthermore, audiologists can play an active role in multidisciplinary HIV care teams by providing updates on auditory health risks, contributing to patient management plans and advocating for routine ototoxicity monitoring protocols. Such training would facilitate prompt referrals to audiologists and enable early interventions. This training can be coordinated by qualified audiologists and/or training institutions as part of their graduate student training programmes. Incorporating audiology modules into healthcare curricula and developing continuous professional development workshops for HIV care providers can ensure sustainable knowledge transfer. Additionally, incorporating strategies from oncology-focused audiological programmes, such as educating healthcare providers on ototoxic risk factors and optimising follow-up care protocols, can enhance the effectiveness of EHDI initiatives for HEU children. These programmes highlight the value of a cohesive framework where audiologists actively contribute to both preventive and therapeutic care, ensuring comprehensive support for high-risk paediatric populations. Thirdly, *developing targeted health communication strategies* should form part of the South African Speech-Language and Hearing Professions core mandate and scope of function. Educating mothers, particularly in low-resource communities, on the importance of hearing assessments is crucial. By raising awareness about the specific hearing risks associated with HIV and ART, healthcare providers can empower mothers to seek routine hearing assessments for their children. Fourthly, *expanding research into ART ototoxicity* should form part of the country’s research agenda. Future research should focus on several critical areas, including longitudinal studies on the cumulative effects of ART regimens on auditory health, the exploration of protective interventions against ototoxicity, and the integration of advanced diagnostic technologies for early hearing loss detection in HEU children. Such research would provide the evidence base needed to inform clinical guidelines and healthcare policies. Additionally, to implement the level of changes proposed, a task team comprising diverse stakeholders – such as audiologists, infectious disease specialists, public health officials, policymakers and community representatives – should be established. This task team could guide the development, implementation and monitoring of EHDI programmes tailored for HEU children, ensuring that these initiatives are contextually relevant and sustainable within South Africa’s resource-limited settings. While ART remains a vital intervention, further research into the its ototoxic side effects is necessary. Understanding which drugs pose the highest risks to auditory health can guide treatment choices and may even influence the development of new, less ototoxic drugs. Future research should also prioritise evaluating the specific auditory risks associated with commonly used ART drugs such as NVP. This includes investigating the cumulative effects of exposure and identifying potential protective measures or alternative treatment options to minimise ototoxicity in HEU children. Lastly, *enhancing data collection and monitoring for longitudinal studies* should form part of electronic health records platforms developments and big data use and analysis training. The implementation of a robust data collection and tracking system for hearing screenings in HEU children can inform future research and allow for a better understanding of the long-term outcomes of HIV and ART exposure on auditory health.

## Conclusion

Targeted interventions, including routine EHDI programmes, integration of audiologists into paediatric care, and policy-level commitments to address ART ototoxicity, are vital for reducing the burden of hearing loss among HEU children in South Africa. Audiologists play an indispensable role in early detection and intervention, yet their involvement remains limited in many public health settings in South Africa. Given the documented risks to hearing health in HEU children, an effective EHDI programme that integrates routine audiological screenings within postnatal HIV care is urgently needed. The growing body of evidence from diagnostic studies in South Africa and globally reinforces the need to categorise HEU children as a priority group for routine hearing assessments. Findings from studies by Khoza-Shangase and Nesbitt (2023) and Padilha et al. (2018) highlight that early identification of hearing loss in this population is essential not only for mitigating developmental delays but also for informing healthcare policies targeting vulnerable paediatric groups. Such a programme not only aligns with South Africa’s commitments to equitable healthcare but also addresses the country’s public health burden associated with HIV by ensuring that HEU children have the best chance at achieving their developmental potential. To ensure sustainable and impactful contributions, audiologists must remain informed about evolving ART regimens and their auditory side effects. Active engagement in research, policy discussions, and interdisciplinary healthcare teams will empower audiologists to provide proactive, contextually relevant care in addressing the unique challenges faced by HEU children. The inclusion of audiologists in multidisciplinary healthcare teams and the integration of routine hearing screenings in HIV-related paediatric care are essential steps towards comprehensive, preventive healthcare that will benefit both individuals and society at large. Incorporating successful elements from programmes addressing cancer-related ototoxicity, such as systematic audiological tracking and multidisciplinary collaboration, can serve as a benchmark for improving EHDI services for HEU children. By adopting these evidence-based practices, South Africa’s healthcare system can strengthen its approach to addressing hearing loss in this vulnerable population. The proposed changes to prioritise the auditory health of HEU children require coordinated efforts across multiple sectors. Establishing a national task team would be an effective strategy to align stakeholders, streamline resources and implement robust EHDI programmes. Future research should also focus on refining these programmes by addressing gaps in knowledge, particularly around ART-related ototoxicity and innovative approaches to preventive audiology.
